# Enhancement of cellulase production in *Trichoderma reesei* RUT-C30 by comparative genomic screening

**DOI:** 10.1186/s12934-019-1131-z

**Published:** 2019-05-10

**Authors:** Pei Liu, Aibo Lin, Guoxiu Zhang, Jiajia Zhang, Yumeng Chen, Tao Shen, Jian Zhao, Dongzhi Wei, Wei Wang

**Affiliations:** 1 State Key Lab of Bioreactor Engineering, East China University of Science and Technology, P.O.B. 311, 130 Meilong Road, Shanghai, 200237 China; 2 Sunson Industry Group Co, Ltd, Beijing, China

**Keywords:** *Trichoderma reesei*, RUT-C30, Genome sequencing, Cellulase production, *tre108642*, *tre56839*, Alcohol dehydrogenase, CRE1

## Abstract

**Background:**

Cellulolytic enzymes produced by the filamentous fungus *Trichoderma reesei* are commonly used in biomass conversion. The high cost of cellulase is still a significant challenge to commercial biofuel production. Improving cellulase production in *T. reesei* for application in the cellulosic biorefinery setting is an urgent priority.

**Results:**

*Trichoderma reesei* hyper-cellulolytic mutant SS-II derived from the *T. reesei* NG14 strain exhibited faster growth rate and more efficient lignocellulosic biomass degradation than those of RUT-C30, another hyper-cellulolytic strain derived from NG14. To identify any genetic changes that occurred in SS-II, we sequenced its genome using Illumina MiSeq. In total, 184 single nucleotide polymorphisms and 40 insertions and deletions were identified. SS-II sequencing revealed 107 novel mutations and a full-length wild-type carbon catabolite repressor 1 gene (*cre1*). To combine the mutations of RUT-C30 and SS-II, the sequence of one confirmed beneficial mutation in RUT-C30, *cre1*_96_, was introduced in SS-II to replace full-length *cre1*, forming the mutant SS-II-*cre1*_96_. The total cellulase production of SS-II-*cre1*_96_ was decreased owing to the limited growth of SS-II-*cre1*_96_. In contrast, 57 genes mutated only in SS-II were selected and knocked out in RUT-C30. Of these, 31 were involved in *T. reesei* growth or cellulase production. Cellulase activity was significantly increased in five deletion strains compared with that in two starter strains, RUT-C30 and SS-II. Cellulase production of *T. reesei* Δ108642 and Δ56839 was significantly increased by 83.7% and 70.1%, respectively, compared with that of RUT-C30. The amount of glucose released from pretreated corn stover hydrolyzed by the crude enzyme from Δ108642 increased by 11.9%.

**Conclusions:**

The positive attribute confirmed in one cellulase hyper-producing strain does not always work efficiently in another cellulase hyper-producing strain, owing to the differences in genetic background. Genome re-sequencing revealed novel mutations that might affect cellulase production and other pathways indirectly related to cellulase formation. Our strategy of combining the mutations of two strains successfully identified a number of interesting phenotypes associated with cellulase production. These findings will contribute to the creation of a gene library that can be used to investigate the involvement of various genes in the regulation of cellulase production.

**Electronic supplementary material:**

The online version of this article (10.1186/s12934-019-1131-z) contains supplementary material, which is available to authorized users.

## Background

Lignocellulosic biomass, which consists of cellulose, hemicellulose, and lignin, is a renewable resource that is abundantly available for the production of biofuels and chemicals. Biological conversion of lignocellulosic biomass into fermentable sugars by cellulosic enzymes is an environment-friendly and promising approach [1, 2]. However, the production cost of biomass-degrading enzymes is still a significant challenge for commercial biofuel production [2]. *Trichoderma reesei* (an anamorph of *Hypocrea jecorina*) has been widely used to produce commercial cellulase required for the complete hydrolysis of lignocellulose [3]. The cellulase produced by *T. reesei* mainly comprises two cellobiohydrolases (CBHI and CBHII), two endoglucanases (EGI and EGII), and *β*-glucosidase I (BGLI) that synergistically hydrolyze lignocellulosic materials, in concert with related xylanases and auxiliary proteins [1, 3].

In general, the regulation of cellulase expression in *T. reesei* depends on several transcription factors [4]. Xylanase regulator 1 (XYR1) is essential for the expression of most cellulase and xylanase genes [4, 5]. Moreover, expression of cellulase and xylanase genes is subject to carbon catabolite repression (CCR) [6], regulated by carbon catabolite repressor 1 (CRE1) [7]. CCR facilitates preferential assimilation of easily metabolized carbon sources by inhibiting the expression of enzymes involved in the catabolism of other carbon sources. This is essential for the adaptation and survival of *T. reesei* [4, 6].

**Fig. 1:**
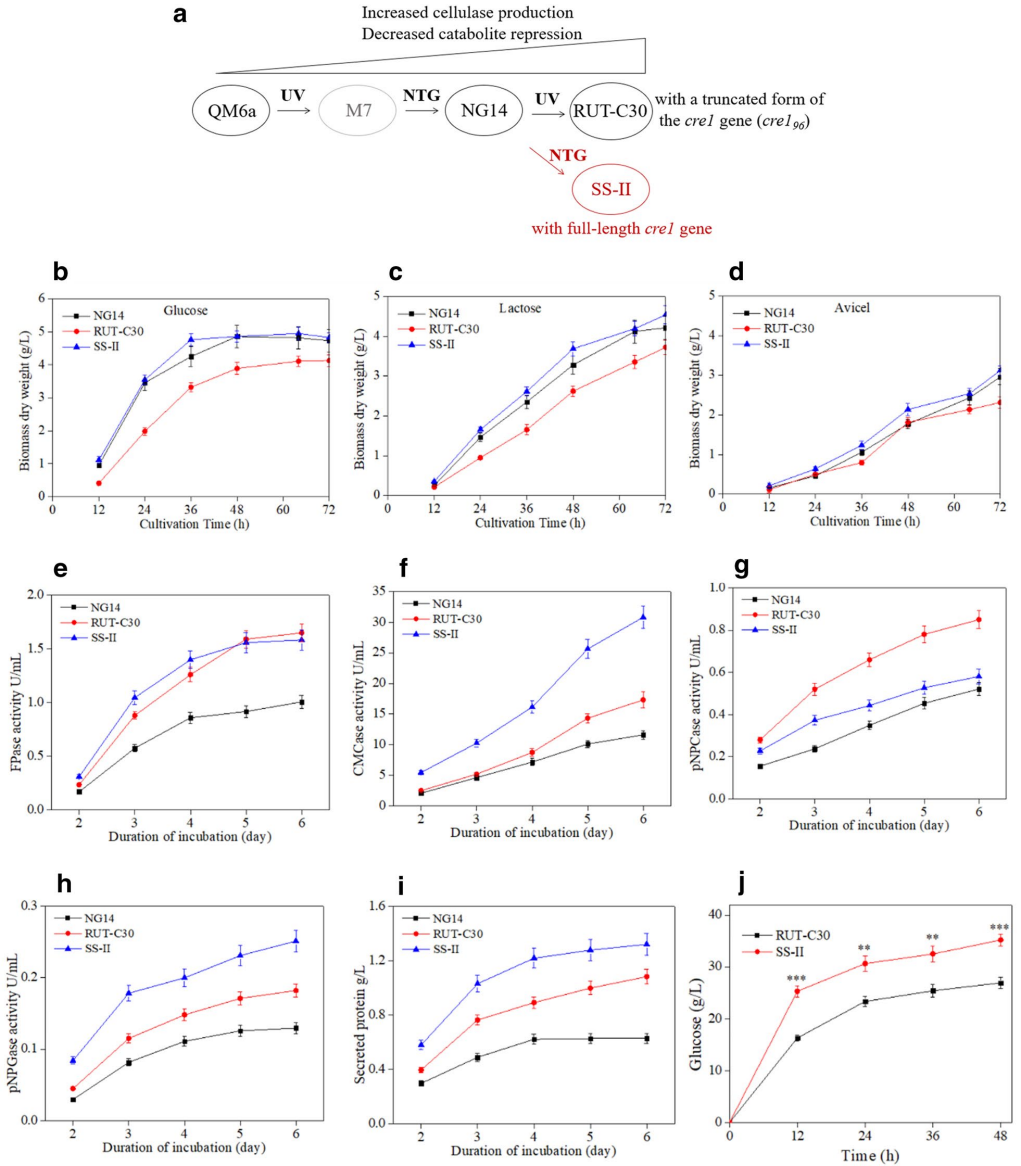
Phenotypic characteristics of SS-II. **a** Cell line of *T. reesei* hyper-cellulolytic mutant SS-II. UV, ultraviolet; NTG, N-nitrosoguanidine. Biomass dry weight of *T. reesei* strains were measured in MA medium containing 2% (w/v) glucose (**b**), 2% (w/v) lactose (**c**), or 2% (w/v) Avicel (D) as the sole carbon source. FPase (**e**), CMCase (**f**), pNPCase (**g**), and pNPGase (**h**) activities and total secreted protein (**i**) of *T. reesei* strains were measured using Avicel as the carbon source. **j** Hydrolysis of pretreated corn stover using the crude enzyme from *T. reesei* strains at 20 FPU/g dry biomass. Values are the mean ± SD of the results from three independent experiments. Asterisks indicate significant differences (*p < 0.05, **p < 0.01, ***p < 0.001, Student’s *t* test)

Classical mutagenesis techniques have been used to generate many *T. reesei* hyper-cellulolytic strains that exhibit increased production of cellulases compared to that in the progenitor strain QM6a [8–12]. There are two distinct pedigree lineages of *T. reesei* mutant strains [8, 11]. One was developed at Rutgers University (Fig. 1a). It includes the NG14 strain, which was derived from strain M7 (no longer available, shown in gray in Fig. 1a) through chemical mutagenesis using *N*-nitrosoguanidine (NTG) [13]. *T. reesei* RUT-C30, a carbon catabolite-repression mutant, was isolated from NG14 using ultraviolet (UV) mutagenesis. RUT-C30 is one of the best cellulase hyper-producers available in the public domain. RUT-C30 produces twice the amount of extracellular protein as that in the parental strain NG14 [8] and has diverse applications in research and industry [2]. Improving cellulase production in *T. reesei* RUT-C30 for application in the cellulosic biorefinery setting is increasingly becoming a focus of research [2, 14, 15].

Genetic changes can influence protein synthesis and secretion in *T. reesei*. Recently, the genomes of several *T. reesei* mutants were analyzed using a variety of techniques and several mutation sites were reported [11, 12, 16–18]. Genome sequencing of RUT-C30 and its parental strain NG14 revealed 126 single nucleotide polymorphisms (SNPs) and 22 insertions and deletions (indels) between the original strain QM6a and NG14 mutant strain, and 97 SNPs and 11 indels between NG14 and RUT-C30 [11]. To date, two mutations involved in cellulase production in RUT-C30 have been identified. One is a truncated form of the *cre1* gene (*cre1*_*96*_), which exerts a positive regulatory influence on the expression of target genes [19–21]. The other is a frameshift mutation in the glucosidase II alpha subunit gene [22]. Interestingly, a *cre1* mutation was also found in another *T. reesei* hyper-cellulolytic mutant, PC-3-7 [23]. Mutations of *cre1* have been consistently linked to the hyper–production of cellulase. Many other mutations have been found in other cellulase hyper-producers, usually accompanied by the identification of novel genes that are involved in cellulase production. A comparative genomic analysis of PC-3-7 and its parent KDG-12 revealed a novel beta-glucosidase regulator BglR with a missense mutation. The deletion of BglR contributed to improved cellulase production [24]. Pei et al. [25] analyzed the hypersecretion-related mutant genes in *T. reesei* RUT-C30 and systematically deleted their orthologs in the fungus *Neurospora crassa* to identify several genes related to cellulase production, particularly *Ncap3m*. The deletion of *Ncap3m* increased the filter paper activity (FPase) and lignocellulose secretion by approximately 44% and 50%, respectively [25]. The novel transcription factor *vib1* was found by genome sequencing analysis of *T. reesei* QM9978 [17]. Zhang et al. improved cellulase production of *T. reesei* RUT-C30 by overexpressing *vib1* [14]. Therefore, genome-wide analysis of mutations is an efficient approach for the identification of novel genes involved in cellulase production.

In addition to the aforementioned genes, other genes that affect cellulase expression in *T. reesei* have also been widely studied. They include genes encoding transcription activators (ACE2 [26], ACE3 [27], CRZ1 [28], and Hap2/3/5 [29]); transcription repressors (ACE1 [30], Pac1 [31], and Rce1 [32]); ß-importin KAP8 [33]; Ndt80-like transcription factor RON1 [34]; methyltransferase LAE1 [35]; sugar transporters (Stp1 [36] and Crt1 [36]; and TrSTR1 [37]). The data are important for understanding the mechanism of cellulase regulation and expression in *T. reesei*.

In this study, we sequenced a *T. reesei* hyper-cellulolytic mutant (donated by Sunson Industry Group Co., Ltd, China) designated SS-II, which has been used for industrial production and has not been characterized through academic research. It exhibits a remarkable increase in the growth rate and carboxymethyl cellulase (CMCase) and p-nitrophenyl-β-d-glucosidase (pNPGase) activity compared with those of RUT-C30. SS-II also shows improved ability to degrade lignocellulosic biomass. To identify novel genes related to cellulase production and to acquire more efficient cellulase-producing strains, 57 genes that were mutated only in SS-II and which have been demonstrated to be involved in transport, secretion, protein metabolism, and transcription were deleted in *T. reesei* RUT-C30 to construct mutants to screen their effects on cellulase production. Genome sequencing provided insight into the influence of genetic changes in cellulase production, ultimately leading to the production of more efficient cellulase-producing strains. The findings indicate that introducing specific mutations from one hyper-cellulolytic strain into another is a feasible strategy to improve cellulase production.

## Results

### Phenotypic characteristics of *T. reesei* hyper-cellulolytic mutant SS-II

We obtained the *T. reesei* hyper-cellulolytic mutant strain, SS-II, which was used for cellulase production, from Sunson Industry between 2002 and 2010. was derived from strain NG14 through NTG mutagenesis. The well-known hyper-cellulolytic mutant RUT-C30 (ATCC 56765) was also isolated from NG14 using UV mutagenesis (Fig. 1a).

To characterize the differences in growth rates between SS-II and RUT-C30, *T. reesei* strains were grown in MA medium with 2% (w/v) glucose, 2% (w/v) lactose, or 2% (w/v) Avicel as the sole carbon source. SS-II grew significantly faster than RUT-C30 on all three carbon sources (Fig. 1b–d). The genetic basis underlying the high growth rate of the SS-II requires further study.

To characterize the cellulase productivity of SS-II, *T. reesei* strains were grown containing 2% (w/v) Avicel as the carbon source. SS-II exhibited levels of FPase activity similar to that of the hyper-cellulolytic mutant RUT-C30 (Fig. 1e). SS-II exhibited lower pNPCase activity than RUT-C30. However, CMCase activity, pNPGase activity, and extracellular protein production in SS-II were higher than those in RUT-C30 (Fig. 1f–i). SS-II, as a *T. reesei* hyper-cellulolytic mutant strain, also exhibited considerably enhanced CMCase, pNPCase, and pNPGase activities compared with those of NG14 (Fig. 1e–i).

When the same FPase loading (20 filter paper cellulase units [FPU]/g dry biomass) was used to hydrolyze pretreated corn stover (PCS), SS-II showed an increase of 30.9% in glucose concentration, reaching 35.2 g/L, compared to that of RUT-C30 (Fig. 1j). The enhanced pNPGase and CMCase activities in the cellulase set of SS-II accounted for the higher glucose yield than that of RUT-C30 when the same FPase loading was used (Fig. 1j). Cellulase produced by SS-II was more effective than that from RUT-C30 for the degradation of lignocellulosic biomass to produce glucose. The difference in the ratios of the four kinds of hydrolase activities in the cellulase set of SS-II compared with those of RUT-C30 indicated again that the genetic basis of SS-II was quite different from that of RUT-C30, although SS-II and RUT-C30 were both derived from the same parental strain NG14.

### Genomic analysis of *T. reesei* SS-II

The SS-II genome was sequenced to identify the genetic differences in SS-II and explore new genetic features that may be involved in the hyper-production of cellulase. The sequence determined in the Whole Genome Shotgun project has been deposited at DDBJ/ENA/GenBank under the accession number SIJN00000000. The version described in this paper is version SIJN01000000.

In total, 8,232,344 paired-end reads (98.05% of the total) were mapped to the wild-type (QM6a) reference genome, representing an average coverage depth of 52×. This resulted in 97.02% coverage of the QM6a reference genome. Using the quality filter described in the methods, 184 single nucleotide polymorphisms (SNPs) and 40 insertions/deletions (indels) were identified in the SS-II genome (Additional file 1). The majority of the SNPs (71%) were in non-coding regions, largely in the promoter region. Forty-one (1.3%) of the SNPs were in the exons. Of these, 26 SNPs caused a change in the amino acid sequence. Most indels (80.8%) were also in the non-coding regions, with only three occurring in the exons (Table 1).

**Table 1:** Number of genes affected by different SNPs and indels

Total SNPs	184 (77)^b^	Total indels	40 (30)^b^
In promoters	60 (18)	In promoters	8 (5)
In terminators	33 (22)	In terminators	13 (11)
In introns	13 (5)	In introns	5 (5)
In exons	41 (20)	In exons	3 (1)
Synonymous	15 (4)	Total intergenic hits^a^	26
Nonsynonymous	26 (13)	Number of genes affected^a^	27
Total intergenic hits^a^	131		
Number of genes affected^a^	130		

^a^The difference in the sum is due to mutations affecting the same gene simultaneously at the two regions—promoter and terminator. Differences in genes affected are due to several single nucleotide exchanges occurring in the same gene

^b^SNPs and indels that exist in SS-II, but not in NG14 and RUT-C30

Of the 184 SNPs detected in the genome of SS-II, 77 were unique to this strain. They were not inherited from the parental strain NG14 and did not exist in the genome of the RUT-C30 offspring of NG14 [11]. Of the 40 indels detected in SS-II, 30 were unique to SS-II. Among the mutated genes affected by the 77 SNPs and 30 indels, 57 genes (Table 2) were obtained based on whether the SNPs and indels were located in the exon, promoter (within 1 kb upstream of the start codon), terminator (within 0.5 kb downstream of the stop codon), or intron regions. These 57 selected genes were affected by the unique SNPs and indels, which were present in SS-II, but not in NG14 or RUT-C30.

**Table 2:** List of FPase activities produced by *T. reesei* RUT-C30 mutants for genes affected by SNPs and indels in *T. reesei* SS-II

No.	Trire2: protein ID	Mutation (element: amino acid change)	Annotation/function	FPase activity (U/mL) of KO strains^a^	Increased vs RUT-C30 (%)^b^	*t*-test^c^
1	108642	A → G (exon: synonymous variant)	Unknown protein	3.05 ± 0.16	+ 83.7%	***
2	56839	T → A (promoter)	Zinc-dependent alcohol dehydrogenase	2.82 ± 0.14	+ 70.1	***
3	108784	− 1: A (intron)	Alcohol dehydrogenase GroES	2.55 ± 0.13	+ 53.4	***
4	66256	T → C (terminator)	NAD(P)-binding protein	2.45 ± .15	+ 47.3	**
5	74622	− 16: GATGACGA TGATTTTC (terminator)	P-loop containing nucleoside triphosphate hydrolase protein	1.95 ± 0.078	+ 17.3	*
6	108914	T → C (exon: Cys_193_ → Arg)	Methyltransferase type 11	1.88 ± 0.11	+ 13.3	–
7	109925	A → G (promoter)	Unknown protein	1.81 ± 0.11	+ 9.1	–
8	67504	A → T (terminator)	Conidiospore surface protein	1.80 ± 0.14	+ 8.8	–
9	123441	A → T (promoter)	Unknown protein	1.78 ± 0.11	+ 7.3	–
10	45598	T → C (terminator)	Unknown protein	1.75 ± 0.15	+ 5.3	–
11	23171	T → A (exon: Phe_14759_ → Leu)	AMP-dependent synthetase and ligase	1.75 ± 0.09	+ 5.2	–
12	63558	− 1: A (terminator)	PLC-like phosphodiesterase	1.70 ± 0.13	+ 2.5	–
13	75568	A → T (terminator)	Thioredoxin	1.70 ± 0.12	+ 2.3	–
14	55105	− 12: ACAATGACATG (promoter)	Amylolytic gene expression activator	1.68 ± 0.12	+ 1.3	–
15	109305	G → A (promoter)	Unknown protein	1.64 ± 0.098	− 1.2	–
16	109304	G → A (promoter)	Unknown protein	1.62 ± 0.11	− 2.2	–
17	104898	A → T (exon: Asp_227_ → Val); A → T (exon: Gln_229_ → Leu); G → T (exon: Gly_230_ → Val) A → T (exon: Gln_233_ → Leu)	Unknown protein	1.62 ± 0.12	− 2.3	–
18	67806	A → T (exon: Ile_120_ → Asn)	Amino acid transporter	1.61 ± 0.086	− 2.8	–
19	120806	− 1: C (intron); − 2: AG (terminator); − 1: T (terminator)	Pkinase-domain-containing protein	1.60 ± 0.095	− 3.5	–
20	120044	T → G (promoter)	GDP dissociation inhibitor	1.59 ± 0.10	− 4.3	–
21	56934	T → G (promoter)	Carbohydrate kinase	1.58 ± 0.095	− 4.6	–
22	43161	C → G (terminator)	Unknown protein	1.58 ± 0.094	− 4.8	–
23	62053	+ 1: T (promoter)	YVTN repeat-like/Quino protein amine dehydrogenase	1.58 ± 0.11	− 5.2	–
24	110688	− 1: G (intron)	Unknown protein	1.56 ± 0.13	− 5.8	–
25	76453	A → G (exon: Met_867_ → Thr)	ABC transporter	1.54 ± 0.096	− 7.3	–
26	70973	− 3: TAC (terminator)	Acyl-CoA *N*-acyltransferase	1.52 ± 0.075	− 8.3	–
27	66888	− 6: GCAGCA (terminator)	Glycosyltransferase	1.51 ± 0.078	− 8.7	–
28	107297	T → C (promoter)	Unknown protein	1.50 ± 0.088	− 9.2	–
29	107743	T → G (promoter)	Unknown protein	1.48 ± 0.096	− 11.0	–
30	70859	A → G (terminator)	Amidase signature enzyme	1.44 ± 0.098	− 13.1	–
31	75012	G → C (promoter)	Root hair defective 3 GTP-binding protein	1.43 ± 0.094	− 13.8	–
32	63464	− 1: A (intron)	Unknown protein	1.34 ± 0.067	− 19.5	**
33	59381	C → T (promoter)	Class I *S*-adenosyl-l-methionine-dependent methyltransferase	1.28 ± 0.055	− 22.6	**
34	73678	A → C (promoter)	Calnexin	1.20 ± 0.071	− 27.7	**
35	120661	T → C (intron)	Adaptor protein complex AP-1 small subunit	1.17 ± 0.058	− 29.3	**
36	122630	G → A (exon: Ser_140_ → Asn)	Unknown protein	1.16 ± 0.065	− 30.1	**
37	112034	A → G (exon: Phe_122_ → Ser)	MFS general substrate transporter	1.15 ± 0.058	− 30.8	***
38	53811	G → A (exon: Ser_73_ → Leu)	Clathrin adaptor complex	1.15 ± 0.047	− 31.0	***
39	76505	A → T (promoter)	Stress response element binding protein	0.94 ± 0.045	− 43.6	***
40	121915	T → A (intron)	Translationally controlled tumor protein	0.92 ± 0.054	− 44.3	***
41	60887	C → G (exon: His_438_ → Asp)	Unknown protein	0.91 ± 0.038	− 45.4	***
42	81043	G → A (intron)	Zinc finger, TFIIS-type	0.76 ± 0.040	− 54.5	***
43	80339	T → A (terminator); T → G (terminator); T → G (terminator); − 1: T (terminator)	Apolipophorin-III and similar insect proteins	0.72 ± 0.038	− 56.5	***
44	3529	− 1: T (promoter)	Alpha and gamma adaptin binding protein p34	0.68 ± 0.041	− 59.3	***
45	111216	C → T (promoter)	Class II Histone H3 methyltransferase	0.58 ± 0.033	− 65.2	***
46	55868	+ 1: C (terminator)	serine/threonine phosphatase	0.56 ± 0.025	− 66.3	***
47	58191	C → T (promoter)	RAM signaling pathway protein	n.t.		
48	120794	A → G (promoter)	ubiquitin	n.t.		
49	80956	A → C (promoter)	unknown protein	n.t.		
50	124205	G → T (terminator)	RNA polymerase III transcription initiation factor complex	n.t.		
51	74162	+ 3: TCT (terminator)	rRNA helicase RRP3	n.t.		
52	74624	− 16: GATGACGATGATTTTC (terminator)	unknown protein	n.t.		
53	108697	T → G (exon: Ile_49_ → Arg)	Calcium-responsive transcription coactivator	n.t.		
54	102973	+ 3: TCT (exon)	Transcriptional corepressor	n.t.		
55	57217	C → T (exon: synonymous variant)	WD40 repeat-like protein	n.t.		
56	71304	T → C (intron)	Ribosomal protein L39e	n.t.		
57	2125	A → T (intron)	RuvB-like helicase 2	n.t.		

“—” indicates no significant difference

*n.t.* not detected for gene knockout, leading to death

^a^Supernatant of mutant cultures 5 days from cultivation was used to measure FPase activity

^b^The increased percentage of FPase activity relative to the parent strain RUT-C30

^c^Asterisks indicate significant differences (*p < 0.05, **p < 0.01, ***p < 0.001, Student’s *t*-test)

The manner in which the SNPs and indels affected the encoding proteins is summarized in Table 2. The data indicate the types of changes, such as missense mutations or amino acid exchanges. For example, *tre108914* (annotated as methyltransferase type II) was affected by the SNP in the exon, where Cys was changed to Arg owing to the mutation of a T to a C. *tre74622* (annotated as P-loop containing nucleoside triphosphate hydrolase protein) was affected by a deletion of 16 bases “GATGACGATGATTTTC” in the terminator region. Genes were also categorized based on gene ontology (GO) annotation. These 57 genes were probably involved in transport and secretion (9 genes), signal transduction (3 genes), transcription (6 genes), and protein metabolism (18 genes). Mutations in the proteins of SS-II could potentially lead to changes in growth rate and metabolite sensing. Interestingly, several genes containing multiple mutations were found in SS-II. They included *tre76453* with two SNPs and *tre104898* with four SNPs.

### Introduction of truncated form *cre1*_*96*_ sequence from RUT-C30 in SS-II to replace full-length *cre1*

**Fig. 2:**
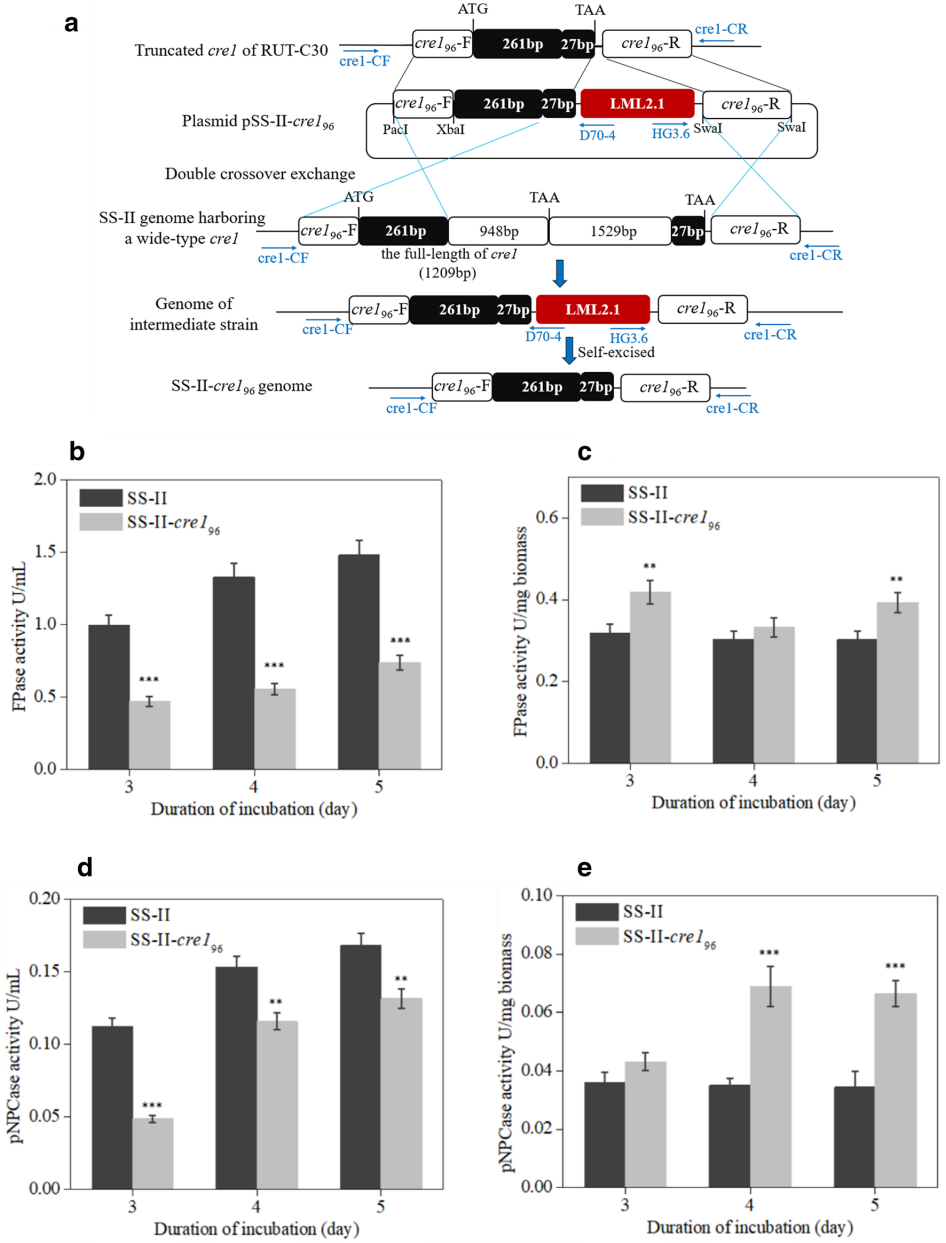
Cellulase production of SS-II-*cre1*_96_. **a** Schematic diagram of the construction of the mutant SS-II-*cre1*_96_. The truncated form of the *cre1*_96_ sequence from RUT-C30 was introduced in SS-II to replace the full-length *cre1* by double crossover exchange. The hygromycin cassette was excised to form SS-II-*cre1*_96_. *T. reesei* strains were cultivated in MA medium using lactose as the carbon source. FPase activity was calculated in two types of units, U/mL (**b**) and IU/mg biomass (**c**). pNPCase activity was also calculated in two types of units, U/mL (**d**) and IU/mg biomass (**e**). Values are the mean ± SD of the results from three independent experiments. Asterisks indicate significant differences (*p < 0.05, **p < 0.01, ***p < 0.001, Student’s *t*-test)

SS-II and RUT-C30 were derived from the same parental strain NG14 (Fig. 1a). Unlike NG14 and the wild-type QM6a, RUT-C30 displays a catabolite-derepressed phenotype in the presence of glycerol or glucose, because of the partial lack of the *cre1* gene (Fig. 2a) encoding the carbon catabolite repressor 1 (CREI) [6, 19, 21]. RUT-C30 harbors a truncated form, *cre1*_96_ (Fig. 2a), which has a positive regulatory influence on the production of cellulase [6, 19, 20]. Genome sequencing revealed that unlike SS-II, which displayed higher cellulase production compared with that of RUT-C30, as measured by FPase, SS-II possesses wild-type *cre1* (Fig. 2a). To address whether the truncated *cre1*_96_ could further improve cellulase production by SS-II and acquire a more efficient cellulase-producing strain, the *cre1*_96_ sequence from RUT-C30 was introduced into SS-II to replace the full-length *cre1*, which generated the mutant SS-II-*cre1*_96_ (Fig. 2a).

The cellulase production of *T. reesei* SS-II-*cre1*_96_ was measured upon cultivation in MA medium with lactose as the carbon source. As shown in Fig. 2b–e, the FPase and pNPCase activities of *T. reesei* SS-II-*cre1*_96_ and SS-II were independently measured as U/mL fermentation broth and U/mg biomass. When the enzyme activities were measured as U/mL fermentation broth (Fig. 2b, d), FPase and pNPCase of *T. reesei* SS-II-*cre1*_96_ were significantly decreased in comparison with those of SS-II. However, when the enzyme activities were measured as U/mg biomass, FPase and pNPCase activities of the SS-II-*cre1*_96_ strain were remarkably increased after 5 days of cultivation compared with those of SS-II (Fig. 2c, e). The obvious difference between U/mL fermentation broth and U/mg biomass of the SS-II-*cre1*_96_ strain was the substantially lower growth rate of SS-II-*cre1*_96_ (Additional file 2: Fig. S1). Nakari-Setälä et al. [20] reported that the deletion of *cre1* increased the quantity of cellulases produced by the wild-type *T. reesei* QM6a strain. Therefore, we mutated *cre1* to obtain a better cellulase-producing strain from SS-II. However, truncation or deletion (Additional file 2: Fig. S2) of *cre1* did not result in higher total cellulase production in SS-II, which was associated with the remarkably lower growth rate. The expression of cellulases in individual hyphae of SS-II-*cre1*_96_ was increased, but total cellulase production of SS-II-*cre1*_96_ was decreased because of the lower growth rate in SS-II-*cre1*_96_. This demonstrated that *cre1* greatly affected the growth of *T. reesei* SS-II and that the full-length *cre1* might be necessary for the rapid growth of SS-II. Mutation of *cre1* is not always the key step to increase total cellulase production in *T. reesei* because of the effect on the growth of *T. reesei*.

### Combining mutations of RUT-C30 and SS-II to produce more efficient cellulase-producing strains from *T. reesei* RUT-C30

The 57 genes (Table 2) with specific mutations in SS-II might be involved in cellulase hyper-production of SS-II. To address whether the 57-gene knockout (KO) could enhance cellulase production in *T. reesei* RUT-C30, we tested the cellulase production capacity of 57 KO mutants of RUT-C30. Furthermore, screening of the KO mutants of RUT-C30 was expected to combine the mutations of both RUT-C30 and SS-II, to acquire more efficient cellulase-producing strains from *T. reesei* RUT-C30.

The 57 genes were knocked out in RUT-C30 (Table 2). Only 46 mutants produced viable homokaryotic colonies, proving that these genes were not essential for survival. Homokaryotic transformants of 11 genes could not be obtained upon deleting them in RUT-C30, suggesting that these were essential genes in RUT-C30 (Table 2). These gene KO mutants died during the spore germination period. Compared with the original strain of RUT-C30, six deletion strains showed severely retarded growth (Additional file 2: Fig. S3).

We further screened these 46 mutants by determining their cellulase production capacity. The measured FPase activities of the mutants 5 days from cultivation in Avicel, which reflected total extracellular cellulase activity [38], are shown in Table 2. The differences in FPase activities of the mutants compared with those of the parental strain RUT-C30 were statistically tested (Table 2).

FPase activities were significantly reduced in 15 strains compared with those in RUT-C30 (Table 2). Among these 15 mutants with reduced FPase activities, deletion of *tre55868* (encoding a serine/threonine phosphatase), *tre81043* (encoding a zinc finger, TFIIS-type), *tre111216* (encoding a histone H3 methyltransferase), and *tre80339* (encoding an apolipophorin-III and similar insect proteins) significantly reduced the FPase activity to 66.3%, 54.5%, 65.2%, and 56.5%, respectively. These 15 genes were speculated to be involved in cellulase hyper-production. However, further studies involving experiments, such as overexpression of these genes in RUT-C30, are required to verify this hypothesis.

Extracellular cellulase production significantly increased in five mutants compared with that of RUT-C30 (Table 2). Of these five mutants, the Δ108642 strain (*tre108642* encodes an unknown protein) displayed an 83.7% increase in FPase activity, which was the highest extracellular cellulase activity observed among the tested strains. The Δ56839 strain (*tre56839* is annotated as a zinc-dependent alcohol dehydrogenase-like protein) demonstrated an approximately 70.1% increase in FPase activity (Table 2). The Δ108642 and Δ56839 mutants displayed over 70% increase in their FPase activities.

**Fig. 3:**
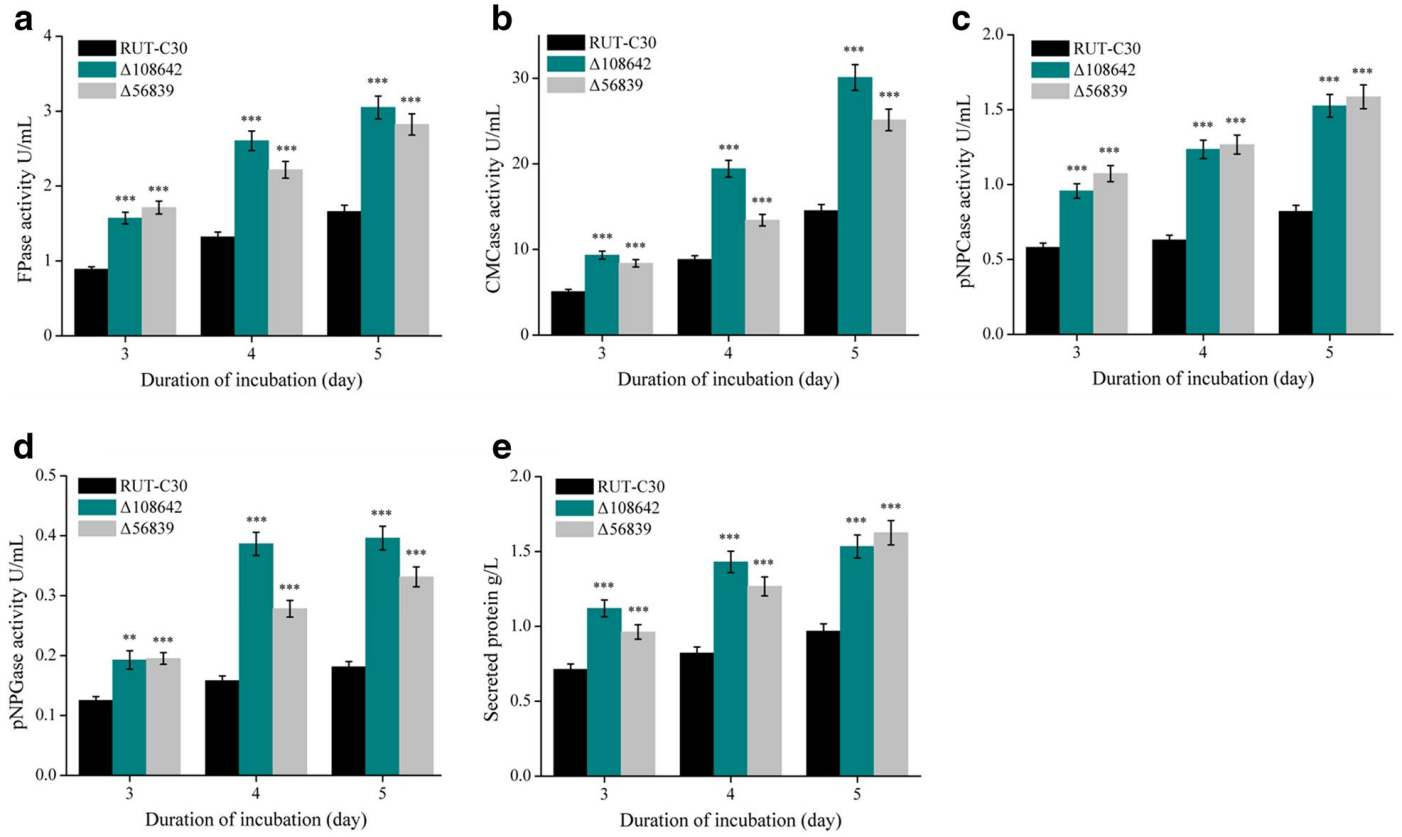
Cellulase activity and secreted protein concentration of *T. reesei* mutants. FPAase (**a**), CMCase (**b**), pNPCase (**c**), pNPGase (**d**) activities, and total secreted protein (**e**) of *T. reesei* strains were measured using Avicel as the carbon source. Values are the mean ± SD of the results from three independent experiments. Asterisks indicate significant differences (*p < 0.05, **p < 0.01, ***p < 0.001, Student’s *t*-test)

To further analyze the effects of deletion of the *tre108642* and *tre56839* genes on the production of the cellulase set, the corresponding activities of various cellulase components and secreted protein concentrations were assayed in Δ108642 and Δ56839. The FPase, CMCase, pNPCase, and pNPGase activities of Δ108642 and Δ56839 were greater than those of the parental strain RUT-C30 (Fig. 3). In agreement with the noticeable increase in cellulase activities, 55% to 70% more secreted proteins were detected in the culture supernatant of Δ108642 and Δ56839 than those of RUT-C30 (Fig. 3). Δ108642 and Δ56839 displayed increased FPase activities and secreted more protein than both the parent strain, RUT-C30 and strain SS-II, which harbored specific mutations (Table 3). The crude enzyme produced by Δ108642 and Δ56839 was used for the hydrolysis of PCS to produce glucose, to evaluate the efficiency of cellulase activity in the *T. reesei* mutants (Table 3) with the same FPase loading (20 FPU/g dry biomass). The glucose yields after enzymatic hydrolysis from *T. reesei* SS-II, Δ108642, and Δ56839 were statistically compared with the yield from *T. reesei* RUT-C30 (Table 3). Compared with that of *T. reesei* RUT-C30, the enzyme set of the Δ108642 mutant showed an 11.9% increase in glucose concentration at 48 h in the PCS hydrolysate. The Δ56839 strain showed a similar glucose concentration in the PCS hydrolysate. Although the PCS hydrolysate efficiency of Δ108642 was lower than that of SS-II, the total cellulase production of Δ108642 was superior to that of SS-II and RUT-C30.

**Table 3:** Cellulase production and hydrolysis of pretreated corn stover by RUT-C30, SS-II, Δ108642, and Δ56839

Strain	FPase activity (U/mL)	Secreted protein (g/L)	Glucose (g/L)^a^	Glucose yield (%)^a^	*t*-test^b^
RUT-C30	1.66 ± 0.08	0.97 ± 0.05	26.9 ± 1.3	66.2 ± 3.2	–
SS-II	1.59 ± 0.10	1.27 ± 0.07	35.2 ± 1.2	86.6 ± 3.3	**
Δ108642	3.05 ± 0.18	1.53 ± 0.09	30.1 ± 1.3	74.1 ± 3.1	*
Δ56839	2.82 ± 0.16	1.62 ± 0.0	27.3 ± 1.1	67.1 ± 3.4	–

“—” indicates s no significant difference

^a^Hydrolysis of pretreated corn stover was performed with FPase loading (20 FPU/g dry biomass) for 48 h

^b^Significance analysis of glucose yield of *T. reesei* SS-II, Δ108642, and Δ56839 relative to *T. reesei* RUT-C30. Asterisks indicate significant differences (*p < 0.05, **p < 0.01, ***p < 0.001, Student’s *t*-test)

### Analysis of Δ108642 and Δ56839 strains with improved cellulase production

The mutants Δ108642 and Δ56839 were also re-complemented by transformation with the vectors R108642 and R56839, respectively. The complementation strains (R108642 and R56839) displayed restorations of the enzyme activities and secreted protein concentration, similar to those of the parent strain RUT-C30 (Additional file 2: Fig. S4). This proved that gene KO of *tre108640* and *tre56839* contributed to the improved cellulase production in Δ108642 and Δ56839.

**Fig. 4:**
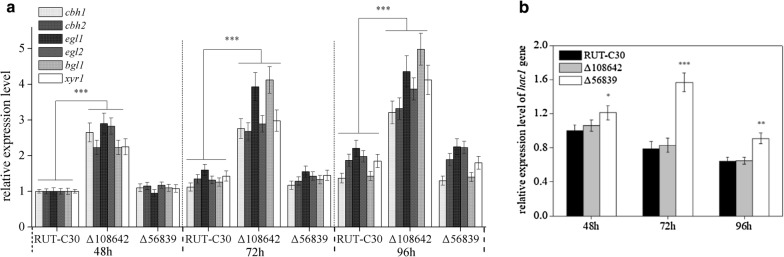
Expression levels of cellulase related genes in *T. reesei* mutants. **a** Gene expression levels were evaluated by qPCR after growth in Avicel for 48, 72, and 96 h. Relative expression levels of *cbh1*, *cbh2*, *egl1*, *egl2*, *bgl1*, and *xyr1*. **b** Relative expression level of *hac1* gene. Data were normalized to the expression of the RUT-C30 at 48 h for each tested gene, and *sar1* expression was used as an endogenous control in all samples. Values are the mean ± SD of the results from three independent experiments. Asterisks indicate significant differences (*p < 0.05, **p < 0.01, ***p < 0.001, Student’s *t*-test)

To further confirm the effects of the mutations on cellulase expression, the transcriptional levels (expression as fold-change) of cellulase related genes, including the five main cellulase genes (*cbh1*, *cbh2*, *egl1*, *egl2*, and *bgl1*), and the key transcriptional activator gene *xyr1*, were analyzed at 48 h, 72 h, and 96 h using RT-qPCR. The mutant Δ108642 exhibited higher expression levels of all five genes compared to RUT-C30 (Fig. 4), consistent with the results of the enhanced cellulase activities. The improved transcription of *xyr1* may be the main reason for the enhanced cellulase expression in *T. reesei* Δ108642 (Fig. 4). The transcript levels of the five main cellulase genes and *xyr1* showed no significant difference between Δ56839 mutant and RUT-C30 (Fig. 4). In addition, the expression level of the activator of chaperone genes (*hac1*) [28] was monitored by RT-qPCR in Δ56839 (Fig. 4). In Δ56839, the *hac1* expression level significantly increased compared with that in RUT-C30 at all time points. The increased expression level of *hac1* activates many genes encoding endoplasmic reticulum chaperones and foldases [28], which might explain the enhanced production of cellulase by Δ56839.

*tre108642* is annotated as an unknown protein, whose function needs further research. Moreover, *tre56839* encodes an alcohol dehydrogenase. To further characterize the function of gene *tre56839*, it was expressed in *Escherichia coli* using the T7 expression vector pET22b. The purified TRE56839 protein was resolved using SDS-PAGE (Additional file 2, Fig. S5). TRE56839 displayed enzyme activity against cinnamyl alcohol. The activity was approximately 13.26/nmol/min/mg using NADP^+^ as the coenzyme (the method of protein expression and enzyme activity assay is shown in Additional file 2). However, the role of *tre56839* in cellulase expression remains unclear. Therefore, the reasons for increased cellulase production by Δ108642 and Δ56839 are different. Scientifically combining the effects of these KOs into one strain needs to be further researched for the construction of more hyper-cellulolytic strains.

## Discussion

In this study, the genetic background of an industrial hyper-cellulase-producing strain of *Trichoderma reesei*, SS-II, was analyzed. *T. reesei* SS-II is derived from *T. reesei* NG-14, the parental strain of the industrial precursor strain RUT-C30. The SS-II strain exhibited higher CMCase activity and increased growth compared with those of the RUT-C30 strain. Cellulase produced by SS-II was more efficient than that from RUT-C30 for the degradation of lignocellulosic biomass to produce glucose. However, the SS-II strain showed similar levels of FPase activity, and lower pNPCase activity compared with those of the RUT-C30 strain. Thus, we sought to combine the mutations of RUT-C30 and SS-II to acquire more efficient cellulase-producing strains.

We sequenced the SS-II strain and identified a number of new mutations, mainly SNPs and indels. Fifty-seven genes that were uniquely mutated in SS-II, but not in NG14 and RUT-C30, were identified. The impact of the 57 genes on cellulase production has not been studied before. The 57 genes were knocked out in RUT-C30. Compared with those in RUT-C30, cellulase activity was significantly increased in five deletion strains. KO of 15 genes in RUT-C30 decreased the FPase activities in RUT-C30. Eleven genes were proven to be essential genes in RUT-C30.

Deletion of *tre108642* in RUT-C30 produced the highest extracellular cellulase activity among the tested strains. The improved transcription of *xyr1* is proposed to be the main reason for the enhanced cellulase expression in *T. reesei* Δ108642. The components of cellulase affected the efficiency of hydrolysis in the pretreated biomass [14, 39]. Cellulase from Δ108642 can produce more glucose through the degradation of PCS than that of *T. reesei* RUT-C30 with the same FPase loading. Strain Δ108642 can produce more cellulase than that of *T. reesei* RUT-C30 or SS-II. *T. reesei* Δ108642 may have potential value in industrial cellulase production. A BLAST search of *tre108642* indicated it encodes an unknown protein (Table 2). The function and mechanism of this protein on cellulase production require further investigation.

Only one alcohol dehydrogenase (glucose-ribitol dehydrogenase 1, GRD1) has been studied in detail in *T. reesei* [40]. The activity of GRD1 on cellobiose was reported. Deletion of *grd1* in *T. reesei* leads to decreased cellulase activity. Presently, we found two alcohol dehydrogenase genes, *tre56839* and *tre108784*, which affected cellulase production in *T. reesei* (Table 2). After examing some substrates (methanol, ethanol, cinnamyl alcohol, butanol, isopropyl alcohol, benzyl alcohol, and ribitol) for TRE56839 and TRE108784, the TRE56839 protein has enzyme activity for cinnamyl alcohol using NADP^+^ as the coenzyme (Additional file 2). However, the catalytic substrates for *tre108784* are still unknown. Cinnamyl alcohol dehydrogenase is a key enzyme in lignin biosynthesis and is involved in the final step of the monolignol synthesis in plants [41, 42]. Genetic evidence indicates that cinnamyl alcohol dehydrogenase deficiency in plants decreases overall lignin and alters cell wall structure [41, 42]. Cinnamyl alcohol dehydrogenases in *Saccharomyces cerevisiae* participate in the degradation of lignin and NADP(H) homeostasis [43]. However, cinnamyl alcohol dehydrogenases in filamentous fungi have not been studied. TRE56839 is the first identified cinnamyl alcohol dehydrogenase in filamentous fungi. We predict that *tre56839*, which encodes the fungal cinnamyl alcohol dehydrogenase, may participate in the degradation of lignin and NADP(H) homeostasis. This ligninolytic activity will be studied. The deletion of *tre56839* may alters the NADP(H) homeostasis in *T. reesei* cells. However, its relationship with increasing protein secretion (Fig. 3) remains unknown. The present data indicate that three NAD(P) related protein genes (*tre56839*, *tre108784*, and *tre66256*) influence cellulase production, as their deletion led to the significant increase of cellulase production (Table 2). The precise mechanism(s) relating NADP(H) homeostasis and cellulase production warrant further investigation.

Among the tested strains, 15 deletion strains showed decreased FPase activities in RUT-C30. Overexpression of the 15 genes may potentially yield cellulase hyper-production strains. Deletion of two putative methyltransferase genes, *tre111216* and *tre59381*, in RUT-C30 significantly decreased cellulase activities (Table 2). Methyltransferases are a large group of enzymes that methylate their substrates [44]. As the most common class of methyltransferases, class I methyltransferase LAE1 (gene ID: *tre41617*, S-adenosyl-methionine-dependent) positively regulates secondary metabolite gene expression in *T. reesei* [35]. Presently, *tre59381* was also BLAST searched and was revealed to encode a class I S-adenosyl-l-methionine-dependent methyltransferase. Therefore, we predict that *tre59381* has a positive effect on the cellulase production, like LAE1. This remains to be confirmed. In addition, *tre111216* was BLAST searched and revealed to encode a histone H3 methyltransferase belonging to class II methyltransferases (Table 2). *Saccharomyces cerevisiae* Set1p is a histone H3 methyltransferase that has a central role in transcriptional regulation and efficient gene expression [45]. Therefore, the *tre111216* deletion in *T. reesei* might affect the transcriptional regulation and reduce cellulase production (Table 2). The deletion strain Δ81043 (*tre81043* is annotated as zinc finger, TFIIS-type) displayed significantly reduced cellulase activity (Table 2). Cellulase expression is tightly regulated by transcription factors in *T. reesei* [26, 27, 29, 30]. Therefore, whether *tre81043* is a positive transcription factor for cellulase expression requires further study. Other deleted strains with reduced cellulase production are also meaningful for the understanding of cellulase regulation. These strains include Δ112034 (*tre112034* is annotated as MFS general substrate transporter); *tre112034* might be an important transporter for carbon sources. The present data helped to identify a number of interesting target genes associated with cellulase production.

Further, we combined the beneficial mutation of RUT-C30 into SS-II to acquire a more efficient cellulase-producing strain. One confirmed beneficial mutation is the lack of the full version of CRE1 (CRE1_96_) in RUT-C30, which exerts a positive regulatory influence on the expression of cellulase genes [20, 46]. However, the *cre1*_96_ replacement did not improve the total cellulase production of SS-II because of the remarkably decreased growth rate (Fig. 2). Unlike the wild-type *T. reesei* QM6a, *T. reesei* hyper-cellulolytic strain RUT-C30 displayed a catabolite-derepressed phenotype in the presence of glycerol or glucose, because of the partial lack of the *cre1* gene (*cre1*_*96*_) encoding the truncated carbon catabolite repressor 1 (CRE1_96_). However, *T. reesei* SS-II possesses wild-type *cre1*. The *cre1*_*96*_ sequence from RUT-C30 was introduced into SS-II to replace the full-length *cre1*, forming the mutant SS-II-*cre1*_*96*_ (Fig. 2a). Additionally, we deleted *cre1* in SS-II to form the mutant SS-II-Δ*cre1* (Additional file 2: Fig. S6). Both SS-II-*cre1*_*96*_ and SS-II-Δ*cre1* strains showed remarkedly decreased growth rates compared with *T. reesei* SS-II. This result is consistent with the description by Nakari-Setälä et al. [20] reporting that deleting or truncating *cre1* in wild-type *T. reesei* QM6a causes a growth delay. Therefore, *cre1* truncation is a possible explanation for the slow growth rate of RUT-C30. Possession of wild-type *cre1* is a possible explanation for the faster growth rate of SS-II. The results highlight that a positive attribute confirmed in one cellulase hyper-producing strain might not always work well in other cellulase hyper-producing strains because of different genetic backgrounds.

Our strategy of combining the mutations of RUT-C30 and SS-II successfully helped to identify a number of interesting phenotypes with regard to cellulase production. Novel genes involved in cellulase production will gradually be revealed, in part with the contribution of our data. Our research contributes to the creation of a library of genes, which can be investigated for their involvement in the regulation of cellulase production. These data will be used to develop new, improved, and more efficient cellulase-producing strains by combining the advantages of hyper-cellulolytic mutants.

## Conclusions

*T. reesei* hyper-cellulolytic strain SS-II derived from NG14 grew faster and more effectively degraded lignocellulosic biomass to produce more glucose than the RUT-C30 strain, which was also isolated from NG14. SS-II genome sequencing revealed a full-length *cre1*. Using comparative genomic analysis, we identified multiple mutations in the SS-II strain. Fifty-seven genes mutated only in SS-II but not in NG14 and RUT-C30 were identified. Among these mutated SS-II genes, the *tre108642* and *tre56839* deletions in *T. reesei* RUT-C30 significantly improved cellulase production and secretion. *T. reesei* Δ108642 produced a higher yield of cellulase and was more effective for the degradation of lignocellulosic biomass to produce glucose than RUT-C30. Therefore, combining mutated genes of two hyper-cellulolytic strains to enhance the cellulase production in *T. reesei* might be an effective strategy for enhancing cellulase production.

## Methods

### Microorganism strains and culture conditions

*Escherichia coli* DH5α strain was used for vector construction and propagation. *Agrobacterium tumefaciens* AGL1 strain was used for the transformation of genes into *T. reesei* strains. The strains used in this research included the original strain *T. reesei* QM6a (ATCC 13631), NG14 (ATCC 56767), RUT-C30 (ATCC 56765), and SS-II. Spores were harvested by cultivating fungus on potato dextrose agar plates (PDA) at 28 °C for 6 days. For genomic DNA extraction, strains were cultivated on MA medium [47] containing 2% glucose as a sole carbon source at 28 °C for 48 h.

To analyze enzyme production, conidia (final concentration 10^6^/mL) of *T. reesei* strains were inoculated into 100 mL of MA medium containing 2% (w/v) Avicel (PH-101; Sigma-Aldrich, St. Louis, MO, USA) in a 500 mL shake flask incubated at 28 °C and 200 rpm for 5 to 7 days [48]. Mycelia were collected at different time intervals and kept frozen at − 80 °C for RNA extraction. The supernatant was used for enzyme assays.

### Fungal growth and biomass assay

For *T. reesei* plate growth assays, the conidia were diluted to 10^7^/mL with sterile water. An equal volume of conidia solution (2 μL) was cultured onto the center of PDA plates for approximately 3 to 7 days at 28 °C.

For *T. reesei* biomass assays, conidia (final concentration 10^6^/mL) of *T. reesei* strains were inoculated into 100 mL of MA medium containing 2% (w/v) Avicel, 2% (w/v) lactose, or 2% (w/v) glucose at 28 °C and 200 rpm for 72 h. The biomass dry weight was measured as previously described [48]. Each experiment was performed in three biological replicates.

### Genomic DNA sequencing and bioinformatics analysis

Genomic DNA was prepared with the TIANamp Yeast DNA Kit (Tiangen, Beijing, China). The integrity of the DNA and the absence of RNA contamination were analyzed using an Agilent 2100 Bioanalyzer and 1% agarose gel electrophoresis. Chromosomal DNA of *T. reesei* SS-II was sequenced using a model 251 PE Illumina MiSeq apparatus at the Shanghai Personalbio Biotechnology facility (Shanghai, China). The genome sequence of the wild-type strain QM6a (version 2.0) was downloaded from the Joint Genome Institute of Department of Energy (USDOE-JGI) website (http://genome.jgi-psf.org/Trire2/Trire2.home.html) and was used as the reference for comparative genomic analysis. Sequence reads were mapped onto the reference sequence using bwa (bwa-0.7.5a) [49] with default parameters. PCR duplicates were removed from the bam files using SAMtools (samtools-0.1.19) [50]. High-quality bam files were produced using GATK (GenomeAnalysisTK- 2.2-15) and CountCovariates, TableRecalibration, RealignerTargetCreator, and IndelRealigner [51].

SNP detection was performed by comparing mapped sequences between the reference and the samples. Loci with heterozygous and homozygous genotypes in a sample different from the reference base were defined as SNPs. We used GATK to detect SNPs and indels. The filtering conditions to remove incorrectly identified SNPs and indels were “QD < 2.0,” “MQ < 40.0,” “FS > 60.0,” “HaplotypeScore > 13.0,” “MQRankSum < -12.5,” “ReadPosRankSum < -8.0,” “DP < 4 or DP > 200,” “10 bp containing 3 or more SNPs;” “QD < 2.0,” “ReadPosRankSum < -20.0,” and “FS > 200.0.” The total number of SNPs and indels was counted and categorized as being located in the promoter region (within 1 kb upstream of the start codon), terminator region (within 500 bp downstream of the stop codon), intron, or exon. They were also characterized as to whether they caused a nonsynonymous amino acid substitution within an exon by using the *T. reesei* filtered gene models from the Joint Genome Institute.

### Mutation confirmation of SS-II

To validate that the newly identified SNPs and indels truly exist in the SS-II genome, 0.5- to 1-kb fragments surrounding each mutation were amplified from the SS-II genomic DNA. These fragments were sequenced directly in an ABI 3730 XL sequencer (Majorbio, Shanghai, China). The primers used for amplification and sequencing are listed in Additional file 3.

### Vector construction and transformation

*Trichoderma reesei* RUT-C30 lacking *tku70* [52] was used as a recipient for all targeted gene knockouts. Deletion cassettes for selected genes were constructed by ligating 0.9 to 1 kb 5′- and 3′-flanks of each gene to the hygromycin resistant plasmid LML2.1 [52], according to our previous study [53]. As shown in Additional file 2: Fig. S6, the upstream fragment was ligated into the PacI- and XbaI-linearized LML2.1 using the ClonExpress™ II One Step Cloning Kit (Vazyme, Nanjing, China). Subsequently, the downstream fragment was inserted into the SwaI site to form the deletion cassettes. Primers for the construction of all gene deletion vectors are presented in Additional file 3. The re-complementation cassettes of the genes were constructed by ligating the whole gene sequences (including the 1.5 kb promoter, gene coding sequence, and 1 kb terminator) to LML2.1. Re-complementation cassettes were transformed into the corresponding gene knockout mutants as previously described [53]. Primers for the construction of re-complementation cassettes are shown in Additional file 3. The vector pSS-II-*cre1*_96_ was constructed by ligating upstream and downstream homologous arms to the hygromycin resistant plasmid LML2.1, as shown in Fig. 2a. The vector pSS-II-*cre1*_96_ was introduced into SS-II, to replace full-length *cre1*, forming the mutant SS-II-*cre1*_96_.

All the constructed cassettes were transformed into *T. reesei* RUT-C30 by *Agrobacterium*-mediated transformation (AMT) [52]. Strains were selected using hygromycin B and cefotaxime on Mandel medium. The hygromycin resistant cassette was self-excised by xylose-induced Cre recombinase [52]. The putative gene disruption mutants generated by double crossover were verified by diagnostic PCR using the primers XX-CF and XX-CR (XX represents the gene name) (Primers shown in Additional file 3). The fragments generated from the genome of transformants by PCR using the primers XX-CF and XX-CR were sequenced to verify the correct transformants.

### Real-time quantitative PCR (RT-qPCR)

RNA extraction, reverse-transcription, and RT-qPCR assay were performed following previously described protocols [53]. The forward and reverse primers are listed in Additional file 3.

### Enzyme activity assays

Filter paper hydrolase (FPase) activity, representing total extracellular cellulase activity, was determined using the 3,5-dinitrosalicylic acid method [54]. Endoglucanase activity (CMCase) was measured using 1% carboxymethylcellulose (CMC, Sigma-Aldrich) in 50 mM sodium acetate buffer (pH 5.0) at 50 °C for 30 min. One unit of activity was defined as the amount of enzyme that produces 1 μmol of reducing sugar per min.

Cellobiohydrolase activity (pNPCase) was determined using *p*-nitrophenol-d-cellobioside (pNPC) as a substrate [53]. *β*-glucosidase activity (pNPGase) was determined using *p*-nitrophenol-d-glucopyranoside (pNPG) as a substrate [14]. The release of *p*-nitrophenol was assessed by measuring absorbance at 405 nm. One unit of enzymatic activity was defined as 1 μmol of *p*-nitrophenol released from the substrate per min.

The concentration of proteins in the supernatant was measured using a commercial protein assay kit (BIO-RAD, Hercules, CA, USA) based on the absorbance at 595 nm.

### Enzymatic hydrolysis of pretreated corn stover (PCS)

Hydrolysis of pretreated corn stover (PCS) was performed as previously described [48]. PCS and biodetoxified corn stover were donated by Professor Jie Bao [55]. Hydrolysis experiments were performed in a flask containing 10% (w/v) PCS as the substrate and FPase loading (20 U/g dry biomass) at 50 °C and pH 5.0 for 48 h. Glucose was analyzed by high performance liquid chromatography using a Sugar-PakTM 1 column (6.5 mm × 300 mm; Waters, Milford, MA, USA) at 70 °C using ultrapure water as the mobile phase at a flow rate of 0.5 mL/min. The method for the calculation of glucose yield was based on a prior study [56]. The experiments were performed in triplicate.

### Statistical analyses

Unless otherwise specified, all experiments were biologically and technically performed in triplicate and statistical tests for significance were determined using a one-way analysis of variance (ANOVA) with SPSS software (version 19.0.0.329).

## Additional files

**Additional file 1: Table S1.** List of SNPs and genomic elements affected in SS-II. **Table S2.** List of indels and genetic elements affected in SS-II.

**Additional file 2: Fig. S1.** Biomass dry weight of *T. reesei* strains. **Fig. S2.** Cellulase production of SS-II-Δ*cre1*. **Fig. S3.** Hyphal growth of *T. reesei* mutants and parental strain RUT-C30. **Fig. S4.** Cellulase activity and secreted protein concentration by *T. reesei* complementation strains R108642 and R56839. **Fig. S5.** TRE56839 protein expressed in *E. coli* using SDS-PAGE. **Fig. S6.** Construction of deletion mutants.

**Additional file 3.** Primers used in this study.

## Data Availability

All data generated or analyzed during this study are included in this published article and its additional files.

## Abbreviations

CMCase: carboxymethylcellulose cellulase
SNPs: single nucleotide polymorphisms
indels: insertion/deletions
UV: ultraviolet
NTG: *N*-nitrosoguanidine
FPase: filter paper activity
GO: gene ontology
KO: knockout
MA: Mandels–Andreotti
qRT-PCR: quantitative real-time PCR
SDS-PAGE: sodium dodecyl sulfate polyacrylamide gel electrophoresis
CCR: carbon catabolite repression
BGLI: *β*-glucosidase I
CRE1: carbon catabolite repression regulator 1
BLASTP: protein basic local alignment search tool
IPTG: isopropyl-β-d-thiogalactopyranoside
ADH: alcohol dehydrogenase
