# Supplementary material for: Enhancement of cellulase production in Trichoderma reesei RUT-C30 by comparative genomic screening
Source: Microb Cell Fact. 2019 May 10;18:81. doi: 10.1186/s12934-019-1131-z (PMC6509817; doi:10.1186/s12934-019-1131-z)
Supplement: Supplementary file 1 — Additional file 1: Table S1. List of SNPs and genomic elements affected in SS-II. Table S2. List of indels and genetic elements affected in SS-II. [file 12934_2019_1131_MOESM1_ESM.docx]

**Additional tables**

**Table S1** **List of SNPs and genomic elements affected in strain SS-II**

| **Position** | **Protein ID** | **Mutation** | **Element** | **Amino acid change** | **Annotation/function** |
| --- | --- | --- | --- | --- | --- |
| 1_616401 | 73678 | A→C | Promoter |  | Calnexin |
| 1_1045635 | 53226 | C→A | Promoter |  | ARM repeat-containing protein |
|  | 53492 | C→A | Intron |  | Unknown protein |
| 1_1108451 | 71304 | T→C | Intron |  | Ribosomal protein L39e |
| 1_2497406 |  | A→G |  |  |  |
| 1_2605513 | 44330 | G→T | Exon |  | Serine/threonine protein kinase |
| 1_2613328 | 53105 | T→C | Promoter |  | DNA polymerase |
|  | 53133 | T→C | Terminator |  | Pentatricopeptide repeat protein |
| 1_2888621 |  | T→G |  |  |  |
| 1_3181166 |  | C→T |  |  |  |
| 1_3233804 |  | A→T |  |  |  |
| 1_3404376 |  | A→G |  |  |  |
| 1_3525497 | 53811 | G→A | Exon | Ser_73_→Leu | Clathrin adaptor complex |
|  | 119960 | G→A | Promoter |  | Unknown protein |
| 1_3648559 |  | T→C |  |  |  |
| 2_80111 | 74570 | C→T | Exon | Gly_189_→Asp | ABC1-domain-containing protein |
| 2_124683 | 103447 | A→G | Terminator |  | Unknown protein |
| 2_216513 |  | C→T |  |  |  |
| 2_409668 | 120044 | T→G | Promoter |  | GDP dissociation inhibitor |
| 2_492557 |  | G→A |  |  |  |
| 2_635952 | 21453 | A→T | Exon |  | Putative ATP-dependent RNA helicase DBP7 |
| 2_767703 | 103653  74765 | C→T  C→T | Promoter  Promoter |  | Unknown protein  Bromodomain-containing protein |
| 2_1157914 | 35768 | T→C | Exon | Leu_325_→Pro | Unknown protein |
| 2_1177869 | 44956 | T→A | Exon |  | MFS transporter |
| 2_1235568 | 120173 | C→A | Intron |  | Protein import receptor MAS20 |
| 2_1559187 | 55417 | C→T | Exon |  | Dynein heavy chain |
| 2_1736605 | 75012 | G→C | Promoter |  | Root hair defective 3 GTP-binding protein |
| 2_1901218 | 75072  75074 | C→T  C→T | Promoter  Terminator |  | Snf7 family protein, possibly involved in vesicular trafficking  Cis-Golgi transport protein particle complex subunit |
| 3_336690 | 104161 | A→T | Promoter |  | Importin beta-5 subunit |
| 3_384162 | 56839 | T→A | Promoter |  | GroES-like protein |
| 3_670969 | 104263 | A→G | Promoter |  | Unknown protein |
| 3_906436 | 45456 | C→T | Intron |  | ARM repeat-containing protein |
| 3_1035387 |  | T→C |  |  |  |
| 3_1126055 | 45512 | G→A | Promoter |  | Unknown protein |
| 3_1423427 | 57178  45598 | T→C  T→C | Exon  Terminator |  | Unknown protein  Unknown protein |
| 3_1449780 | 56934 | T→G | Promoter |  | Carbohydrate kinase |
| 3_1706982 | 75568 | A→T | Terminator |  | Thioredoxin |
| 3_1846706 | 104599  56203 | A→G  A→G | Terminator  Promoter |  | Mandelate racemase  Glycosyltransferase family 76 |
| 4_509873 |  | C→A |  |  |  |
| 4_531603 |  | A→G |  |  |  |
| 4_645223 | 120661 | T→C | Intron |  | Adaptor protein complex AP-1 small subunit |
| 4_854661 | 104898 | A→T | Exon | Asp_227_→Val | Unknown protein |
| 4_854667 | 104898 | A→T | Exon | Gln_229_→Leu | Unknown protein |
| 4_854670 | 104898 | G→T | Exon | Gly_230_→Val | Unknown protein |
| 4_854679 | 104898 | A→T | Exon | Gln_233_→Leu | Unknown protein |
| 4_1275053 | 58191 | C→T | Promoter |  | RAM signaling pathway protein |
| 4_1288657 | 58161 | T→A | Terminator |  | GTPase-activating protein |
| 4_1288658 | 58161 | G→T | Terminator |  | GTPase-activating protein |
| 4_1354224 | 57217 | C→T | Exon |  | WD40 repeat-like protein |
| 4_1361526 | 120794  76073 | A→G  A→G | Promoter  Promoter |  | Ubiquitin  Ribonucleoprotein LSM domain |
| 4_1729373 | 57609 | C→T | Exon | Arg_108_→Cys | Unknown protein |
| 4_1771912 | 3027 | T→C | Exon |  | Pso2 (Snm1) protein family involved in DNA interstrand  crosslink repair |
| 4_1814727 | 37761  40945 | G→T  G→T | Promoter  Intron |  | Unknown protein  Unknown protein |
| 5_716519 |  | C→T |  |  |  |
| 5_727374 |  | C→T |  |  |  |
| 5_1001779 | 58391 | G→A | Exon | Pro_88_→Ser | Unknown protein |
| 5_1086144 | 76453 | A→G | Exon | Met_867_→Thr | ABC transporter |
| 5_1086146 | 76453 | T→G | Exon |  | ABC transporter |
| 5_1204664 | 76505 | A→T | Promoter |  | Stress response element binding protein |
| 5_1312421 |  | A→G |  |  |  |
| 5_1553311 | 121087 | G→A | Promoter |  | Unknown protein |
| 5_1670554 | 58561 | C→T | Promoter |  | MFS permease |
| 6_246104 | 105866 | A→T | Promoter |  | Unknown protein |
| 6_285837 | 105874 | A→T | Intron |  | FAD-dependent oxidase |
| 6_285840 | 105874 | G→A | Intron |  | FAD-dependent oxidase |
| 6_395144 |  | T→A |  |  |  |
| 6_395146 |  | T→C |  |  |  |
| 6_604455 | 121177  121178 | G→C  G→C | Terminator  Terminator |  | Unknown protein  Unknown protein |
| 6_604462 | 121177  121178 | A→G  A→G | Terminator  Terminator |  | Unknown protein  Unknown protein |
| 6_604467 | 121177  121178 | T→C  T→C | Terminator  Terminator |  | Unknown protein  Unknown protein |
| 6_604477 | 121177  121178 | T→G  T→G | Terminator  Terminator |  | Unknown protein  Unknown protein |
| 6_927761 | 59381 | C→T | Promoter |  | S-adenosyl-L-methionine-dependent methyltransferase |
| 6_1023496 | 76859 | G→A | Terminator |  | ARM repeat-containing protein |
| 7_482522 | 3501 | G→A | Promoter |  | Unknown protein |
| 7_614946 |  | A→G |  |  |  |
| 7_614951 |  | A→G |  |  |  |
| 7_772040 | 60243 | T→C | Intron |  | Adaptor protein complex AP-1 medium subunit of clathrin-coated vesicles |
| 8_424132 | 77513 | C→T | Exon | Gln_641_→* | Zn2Cys6 transcriptional regulator |
| 8_446760 | 3718 | G→T | Terminator |  | Predicted amino acid aldolase or racemase |
| 8_520585 | 60887 | C→G | Exon | His_438_→Asp | Unknown protein |
| 8_760525 | 106981 | T→A | Promoter |  | DASH complex, subunit Spc34 |
| 8_1033975 | 107072 | A→C | Terminator |  | Unknown protein |
| 8_1049860 |  | T→A |  |  |  |
| 9_43299 | 121735 | C→T | Exon |  | Glycoside hydrolase family 3 |
| 9_317520 | 107297 | T→C | Promoter |  | Unknown protein |
| 9_335933 |  | G→A |  |  |  |
| 9_335946 |  | G→A |  |  |  |
| 9_844024 | 107475 | T→A | Promoter |  | Unknown protein |
| 9_844025 | 107475 | T→G | Promoter |  | Unknown protein |
| 9_872891 |  | T→A |  |  |  |
| 9_880288 |  | A→G |  |  |  |
| 9_1060271 | 121915 | T→A | Intron |  | Translationally controlled tumor protein |
| 9_1194759 | 107601 | G→A | Promoter |  | Tim10/DDP family zinc finger protein |
| 9_1212249 |  | T→C |  |  |  |
| 10_392001 | 78301 | C→T | Promoter |  | Cation efflux protein |
| 10_406234 | 107743 | T→G | Promoter |  | Unknown protein |
| 10_466417 | 78320 | T→C | Promoter |  | Sulfatase |
| 10_492107 | 122050 | C→T | Promoter |  | NIF-domain-containing protein |
| 10_822926 | 48599 | C→G | Promoter |  | PAF acetylhydrolase family protein |
| 11_544338 |  | T→A |  |  |  |
| 11_893313 |  | T→C |  |  |  |
| 11_893331 |  | T→C |  |  |  |
| 11_893349 |  | T→C |  |  |  |
| 11_893351 |  | T→C |  |  |  |
| 11_893382 |  | G→C |  |  |  |
| 11_893399 |  | T→C |  |  |  |
| 11_893473 |  | G→T |  |  |  |
| 11_1117650 |  | C→T |  |  |  |
| 12_681350 | 63558 | C→G | Terminator |  | PLC-like phosphodiesterase |
| 12_681364 | 63558 | C→T | Terminator |  | PLC-like phosphodiesterase |
| 12_681440 | 63558 | A→G | Terminator |  | PLC-like phosphodiesterase |
| 12_969216 | 108642 | A→G | Exon |  | Unknown protein |
| 13_124537 | 108697 | T→G | Exon | Ile_49_→Arg | Calcium-responsive transcription coactivator |
| 13_199540 |  | A→C |  |  |  |
| 13_367774 |  | A→G |  |  |  |
| 13_845372 | 79405 | A→T | Promoter |  | HET-containing protein, unknown |
| 13_861678 | 108914 | T→C | Exon | Cys_193_→Arg | Methyltransferase type 11 |
| 14_21618 | 122630 | G→A | Exon | Ser_140_→Asn | Unknown protein |
| 14_50086 | 108940 | G→A | Exon | Arg_327_→Cys | Putative cutinase transcription factor |
| 14_658748 | 64866 | C→T | Promoter |  | Thioredoxin-like protein |
| 14_671129 | 64375 | C→A | Exon | Gln_292_→Lys | Glycoside hydrolase family 5 |
| 15_158928 |  | A→G |  |  |  |
| 15_165438 | 65104 | T→A | Exon | Tyr_2269_→Phe | Vacuolar protein sorting-associated protein Vps13 |
| 15_298844 |  | A→T |  |  |  |
| 15_358038 |  | T→C |  |  |  |
| 15_415696 | 109305 | G→A | Promoter |  | Unknown protein |
| 15_458201 | 109320 | A→G | Promoter |  | Unknown protein |
|  | 109321 | A→G | Terminator |  | Unknown protein |
| 15_721460 | 64882 | A→G | Terminator |  | MFS permease |
| 16_53679 | 109432 | A→T | Promoter |  | Unknown protein |
| 16_53680 | 109432 | T→G | Promoter |  | Unknown protein |
| 16_357390 | 22841 | C→T | Exon |  | Unknown protein |
| 16_648564 | 40758 | C→A | Promoter |  | Homocysteine methyltransferase |
| 16_655396 | 50268 | C→T | Promoter |  | NAD-dependent deacetylase |
|  | 109619 | C→T | Exon | Ser_63_→Phe | TBP-binding repressor protein |
| 16_817482 | 4921 | G→T | Promoter |  | C2H2 transcriptional regulator |
| 17_438855 |  | T→C |  |  |  |
| 18_57114 | 109925 | A→G | Promoter |  | Unknown protein |
| 18_109135 | 66256 | T→C | Terminator |  | NAD(P)-binding protein |
| 18_143327 | 80339 | T→A | Terminator |  | NAD(P)-binding protein |
| 18_143328 | 80339 | T→G | Terminator |  | NAD(P)-binding protein |
| 18_143425 | 80339 | T→G | Terminator |  | NAD(P)-binding protein |
| 18_329630 |  | A→G |  |  |  |
| 19_44994 |  | T→C |  |  |  |
| 19_393811 |  | C→T |  |  |  |
| 20_37856 | 66895 | G→T | Exon | Trp_134_→Cys | Ribosomal protein S5 domain 2-like protein |
|  | 5140 | G→T | Promoter |  | Cwf15/Cwc15 cell cycle control protein |
| 20_238366 |  | A→G |  |  |  |
| 20_270364 | 110423 | G→A | Exon | Ala_154_→Thr | CAF1 family ribonuclease |
| 20_340466 | 67030 | C→T | Promoter |  | Unknown protein |
| 20_424726 | 123344 | A→C | Promoter |  | Histidine phosphotransferase |
| 21_158677 | 67504 | A→T | Terminator |  | Conidiospore surface protein |
| 21_315015 | 67350 | C→T | Intron |  | Unknown protein |
| 21_366952 | 123422 | A→C | Promoter |  | GTPase-activating protein |
| 21_573615 | 123441 | A→T | Promoter |  | Unknown protein |
| 22_53935 | 67806 | A→T | Exon | Ile_120_→Asn | Amino acid transporter |
| 22_120130 | 81043 | G→A | Intron |  | Zinc finger, TFIIS-type |
| 22_430693 |  | T→A |  |  |  |
| 22_475328 | 67658 | T→C | Promoter |  | A/G-specific adenine DNA glycosylase |
|  | 5363 | T→C | Terminator |  | DNA glycosylase |
| 23_450326 | 123577 | A→G | Terminator |  | Endoplasmic oxidoreductin |
|  | 67938 | A→G | Promoter |  | NAD(P)-binding protein |
| 23_450345 | 123577 | A→C | Terminator |  | Endoplasmic oxidoreductin |
|  | 67938 | A→C | Promoter |  | NAD(P)-binding protein |
| 23_450346 | 123577 | A→C | Terminator |  | Endoplasmic oxidoreductin |
|  | 67938 | A→C | Promoter |  | NAD(P)-binding protein |
| 24_171486 | 23171 | T→A | Exon | Phe_14759_→Leu | AMP-dependent synthetase and ligase |
| 24_380670 |  | C→A |  |  |  |
| 24_430246 | 5502 | G→A | Exon |  | Aldo/keto reductase |
| 25_167708 | 111216 | C→T | Promoter |  | Histone H3 methyltransferase |
| 25_284474 | 68425 | A→T | Promoter |  | Unknown protein |
| 26_126497 |  | G→A |  |  |  |
| 26_126502 |  | T→C |  |  |  |
| 27_107105 | 111468 | C→T | Exon |  | Actin-like protein |
| 28_29750 | 69437 | C→T | Exon |  | B-type cyclin |
| 28_384711 | 69181 | C→T | Promoter |  | Short-chain dehydrogenases/reductase |
| 29_281808 | 82037 | C→G | Promoter |  | MFS phospholipid transporter Git1 |
| 29_379440 | 111861 | A→G | Intron |  | GPCR, PTH11-type |
| 31_208025 | 112034 | A→G | Exon | Phe_122_→Ser | MFS general substrate transporter |
| 32_40433 | 82153 | G→A | Exon |  | Unknown protein |
| 32_137382 | 70071 | T→C | Exon | Leu_607_→Ser | Zn2Cys6 transcriptional regulator |
| 32_220343 | 43161 | C→G | Terminator |  | Unknown protein |
| 34_5546 | 52520 | C→A | Promoter |  | Cyclin |
| 35_33801 | 112231 | C→T | Exon | Ser_1669_→Asn | Kinesin-like protein |
| 36_3820 |  | G→C |  |  |  |
| 37_26185 | 124172 | A→G | Terminator |  | Protein kinase |
| 39_22252 | 124205 | G→T | Terminator |  | RNA polymerase III transcription initiation factor complex |
| 39_43573 | 6015 | T→G | Promoter |  | DNA-directed RNA polymerase I subunit RPA1 |
|  | 6014 | T→G | Promoter |  | Pre-mRNA-splicing factor 3 |
| 39_53626 |  | T→C |  |  |  |
| 42_30178 | 70859 | A→G | Terminator |  | Amidase signature enzyme |
| 49_24127 | 71037 | T→C | Promoter |  | APH-domain-containing protein |
| 49_27446 |  | G→A |  |  |  |
| 60_12449 |  | T→A |  |  |  |
| 63_10780 |  | T→G |  |  |  |

**Table S2 List of** **indels and genetic elements affected in strains SS-II**

| **Position** | **Protein ID** | **Mutation** | **Element** | **Annotation/function** |
| --- | --- | --- | --- | --- |
| 1_1094095 |  | -1: G |  |  |
| 1_2207744 | 74162  102973 | +3: TCT  +3: TCT | Terminator  Exon | rRNA helicase RRP3  Transcriptional corepressor |
| 1_2408138 | 103031  103032 | -1: G  -1: G | Promoter  Terminator | Unknown protein  Putative NRPS-like enzyme |
| 2_338546 | 74622 | -16: GATGACGATGATTTTC | Terminator | P-loop containing nucleoside triphosphate hydrolase protein |
| 2_1436232 | 103853 | +1: A | Promoter | Mitochondrial carrier |
| 2_1480462 | 55868 | +1: C | Terminator | serine/threonine phosphatase |
| 2_1749696 | 55105 | -12: AACAATGACATG | Promoter | Amylolytic gene expression activator |
| 4_1445170 | 120806 | -1:C | Intron | Pkinase-domain-containing protein |
| 4_1445397 | 120806 | -2:AG | Terminator | Pkinase-domain-containing protein |
| 4_1445415 | 120806 | -1: T | Terminator | Pkinase-domain-containing protein |
| 4_1445425 | 120806 | -1: T | Terminator | Pkinase-domain-containing protein |
| 5_1375817 |  | -1: A |  |  |
| 6_82788 | 105808 | -2: TA | Terminator | Unknown protein |
| 6_1320415 | 3400 | -4: TCCC | Exon | RNA-binding proteins |
| 7_307166 |  | -3: GCA |  |  |
| 7_430672 | 121351 | -1: A | Exon | Glycoside hydrolase family 31 |
| 7_676113 | 3529 | -1: T | Promoter | Alpha and gamma adaptin binding protein p34 |
| 8_989123 |  | +1: G |  |  |
| 9_119176 | 62053 | +1: T | Promoter | YVTN repeat-like/Quino protein amine dehydrogenase |
| 12_681405 | 63464  63558 | -1: A  -1: A | Intron  Terminator | Unknown protein  PLC-like phosphodiesterase |
| 13_320498 |  | +1: G |  |  |
| 13_416148 | 108784 | -1: A | Intron | Alcohol dehydrogenase GroES-like |
| 13_804525 |  | -10: CATCCCCCTG |  |  |
| 14_760555 |  | +1: T |  |  |
| 15_491298 |  | +1: G |  |  |
| 15_626643 |  | -6: GATCGC |  |  |
| 16_386701 | 79960 | -1: A | Promoter | Glycosyl hydrolase family 47 |
| 18_143495 | 80339 | -1: T | Terminator | Apolipophorin-III and similar insect proteins |
| 18_227985 | 123114 | -1: G | Intron | Heat shock protein 90 |
| 19_95463 |  | -1:C |  |  |
| 19_385954 | 80592 | -4: CCCA | Promoter | Hamartin protein |
| 20_271896 |  | +1: G |  |  |
| 20_355675 | 66888 | -6: GCAGCA | Terminator | Glycosyltransferase family 71 |
| 20_490402 |  | -1: C |  |  |
| 22_4322 | 110688 | -1: G | Intron | Unknown protein |
| 22_371000 | 81105 | -1: G | Terminator | Unknown protein |
| 24_380664 |  | +1: T |  |  |
| 26_126493 |  | -2: AT |  |  |
| 27_38971 | 111444  123806 | -1: T  -1: T | Promoter  Promoter | Unknown protein  G-protein coupled receptor protein |
| 46_967 | 70973 | -3: TAC | Terminator | Acyl-CoA N-acyltransferase |
