# Supplementary material for: Enhancement of cellulase production in Trichoderma reesei RUT-C30 by comparative genomic screening
Source: Microb Cell Fact. 2019 May 10;18:81. doi: 10.1186/s12934-019-1131-z (PMC6509817; doi:10.1186/s12934-019-1131-z)
Supplement: Supplementary file 2 — Additional file 2: Fig. S1. Biomass dry weight of T. reesei strains. Fig. S2. Cellulase production of SS-II-Δcre1. Fig. S3. Hyphal growth of T. reesei mutants and parental strain RUT-C30. Fig. S4. Cellulase activity and secreted protein concentration by T. reesei complementation strains R108642 and R56839. Fig. S5. TRE56839 protein expressed in E. coli using SDS-PAGE. Fig. S6. Construction of deletion mutants. [file 12934_2019_1131_MOESM2_ESM.docx]

**Supplementary Methods**

**Protein expression and purification**

Candidate genes were PCR amplified from genomic *T. reesei* DNA and amplified fragments were cloned into the expression vector pET22b (Novagen) using pEASY-Uni Seamless Cloning and Assembly Kit. Correctly produced plasmid was confirmed by DNA sequencing and introduced into chemically competent *E. coli* Transetta (DE3) cells (TransGen, Beijing, China) for protein expression. Recombinants were incubated in TB medium until the OD_600_ reached 0.5−0.6, and then induced with isopropyl-*β*-d-thiogalactopyranoside (IPTG; 0.05 mM final concentration) at 18°C for 18 hours. Cells were harvested and disrupted by sonication. Crude enzyme was purified through Ni-NTA affinity column chromatography (Qiagen GmbH, Hilden, Germany) and Superdex G-200 gel filtration column chromatography (GE Healthcare, Piscataway, NJ, USA). Purified protein was examined with SDS-PAGE.

**Enzyme activity assay**

Alcohol dehydrogenase (ADH) activity was based on monitoring the change in absorbance at 340 nm for ADH [1, 2]. The reaction mixture contained 50 mM Tris-HCl, pH 7.4 in presence of 1 mM NAD^+^ or NADP^+^, 10 mM alcohols (such as cinnamic alcohol) and a certain amount of purified soluble protein or distilled water as control at 30°C for measuring the absorbance at 340 nm.

**Reference**

[1] Koutsompogeras P, Kyriacou A, Zabetakis I. Characterizing NAD-dependent alcohol dehydrogenase enzymes of Methylobacterium extorquens and strawberry (Fragaria x ananassa cv. Elsanta).[J]. Journal of Agricultural & Food Chemistry, 2006, 54(1):235-42.

[2] Munir I, Nakazawa M, Harano K. Occurrence of a novel NADP(+)-linked alcohol dehydrogenase in Euglena gracilis. Comparative Biochemistry & Physiology Part B Biochemistry & Molecular Biology, 2002, 132(3):535-540.

**Supplementary figure captions**

**Fig. S1. Biomass dry weight of *T. reesei* strains.** Values are the mean ± SD of the results from three independent experiments.

**Fig. S2. Cellulase production of SS-II-Δ*cre1*.** The FPase (A) and PNPCase (B) activities of *cre1* deletion strain in SS-II (SS-II-Δ*cre1*) were measured compared with that of *T. reesei* SS-II using lactose as the carbon source. Values are the mean ± SD of the results from three independent experiments. Asterisks indicate significant differences (*p < 0.05, **p < 0.01, ***p < 0.001, Student’s *t-*test).

**Fig. S3.** **Hyphal growth of *T. reesei* mutants and parental strain RUT-C30.** (A), Growth of RUT-C30, Δ76505, Δ120661, and Δ3529 on day 7 with glucose as the carbon source. (B), Measurement of colony diameter of A. (C), Growth of RUT-C30, Δ107743, Δ121915, and Δ75012 on day 4. (D), Measurement of colony diameter of C. Values are the mean ± SD of the results from three independent experiments. Asterisks indicate significant differences (*p < 0.05, **p < 0.01, ***p < 0.001, Student’s *t*-test).

**Fig. S4. Cellulase activity and secreted protein concentration by *T. reesei* complementation strains R108642 and R56839.** FPAase (A), CMCase (B), pNPCase (C), pNPGase (D), total secreted protein (E). Values are the mean ± SD of the results from three independent experiments. Asterisks indicate signifcant diferences (*p < 0.05, **p < 0.01, ***p < 0.001, Student’s *t* test).

**Fig. S5. TRE56839 protein expressed in *E. coli* using SDS-PAGE.** The target band was pointed using black arrow.

**Fig. S6. Construction of deleting mutants.** Schematic representation of the selected genes locus from RUT-C30 and deleting mutants. Deletion cassettes for selected genes were constructed by ligating 0.9 to 1 kb 5′- and 3′-flanks of each gene to the hygromycin resistant plasmid LML2.1.


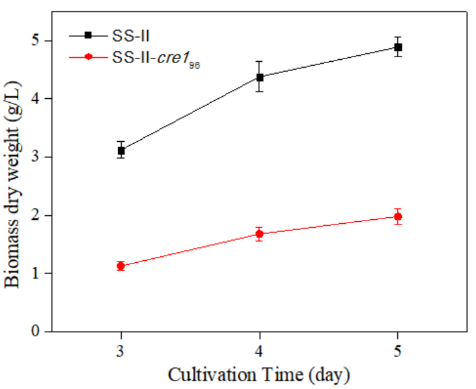


**Fig. S1**


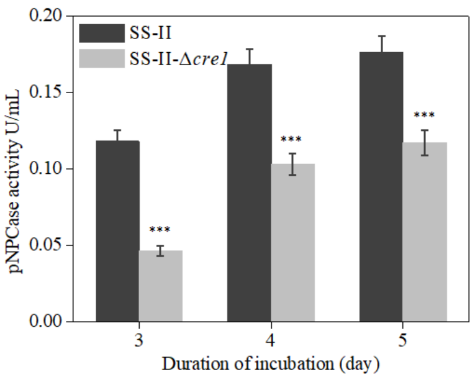

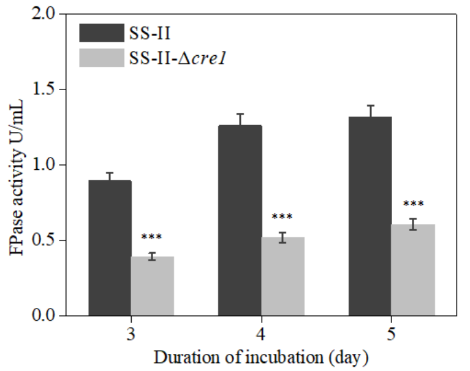
 A B

**Fig. S2**


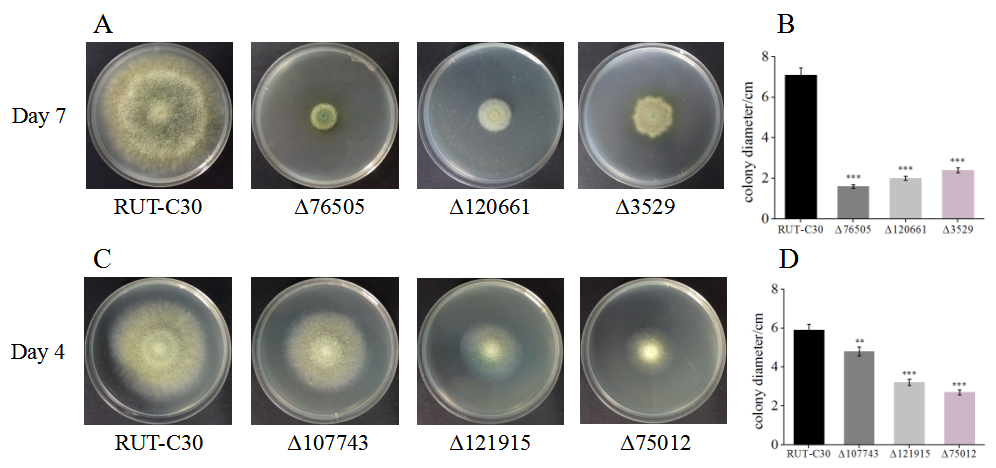


**Fig. S3**

A B C


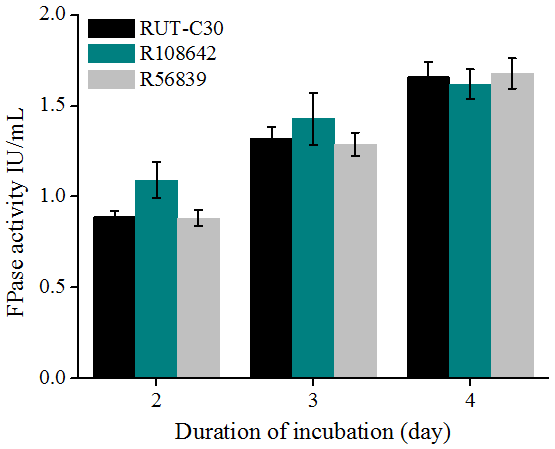

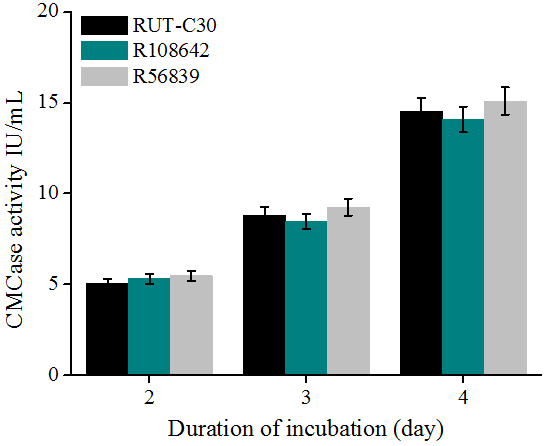

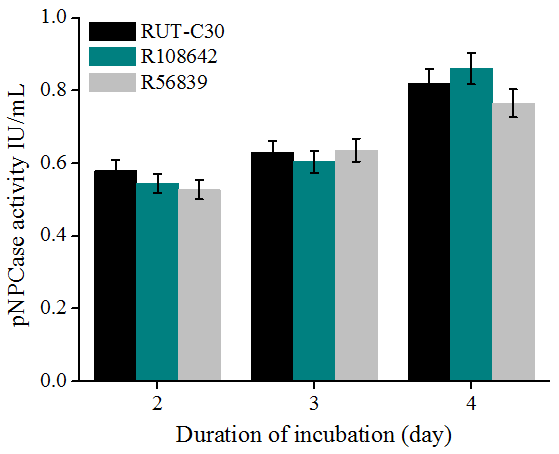


D E


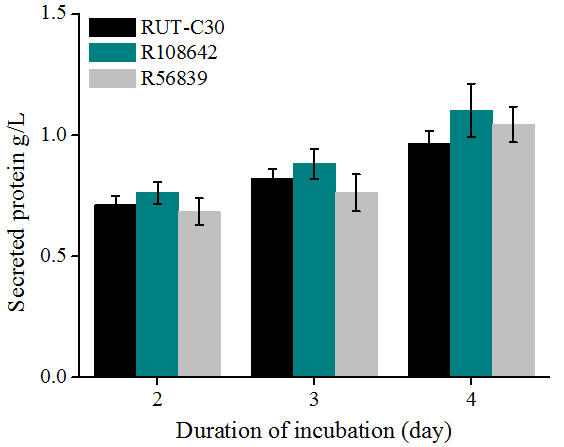

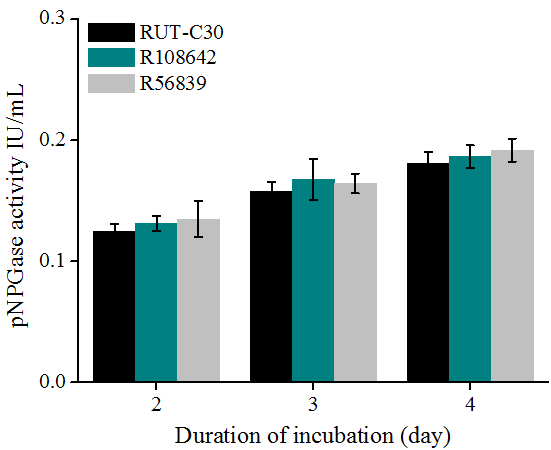


**Fig. S4**


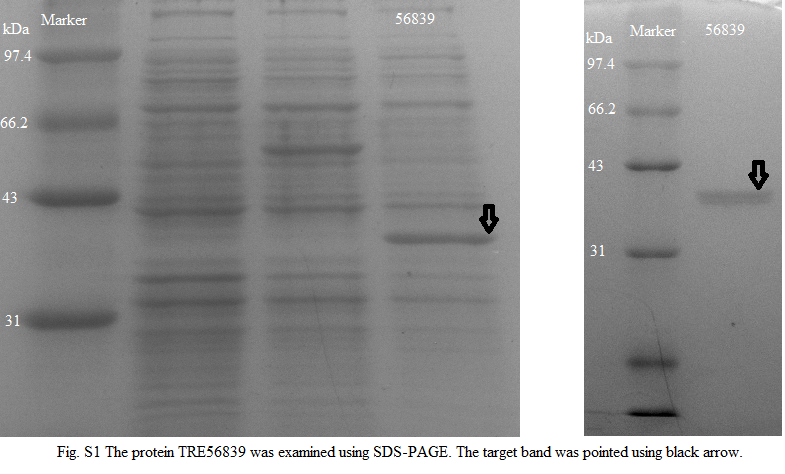


**Fig. S5**


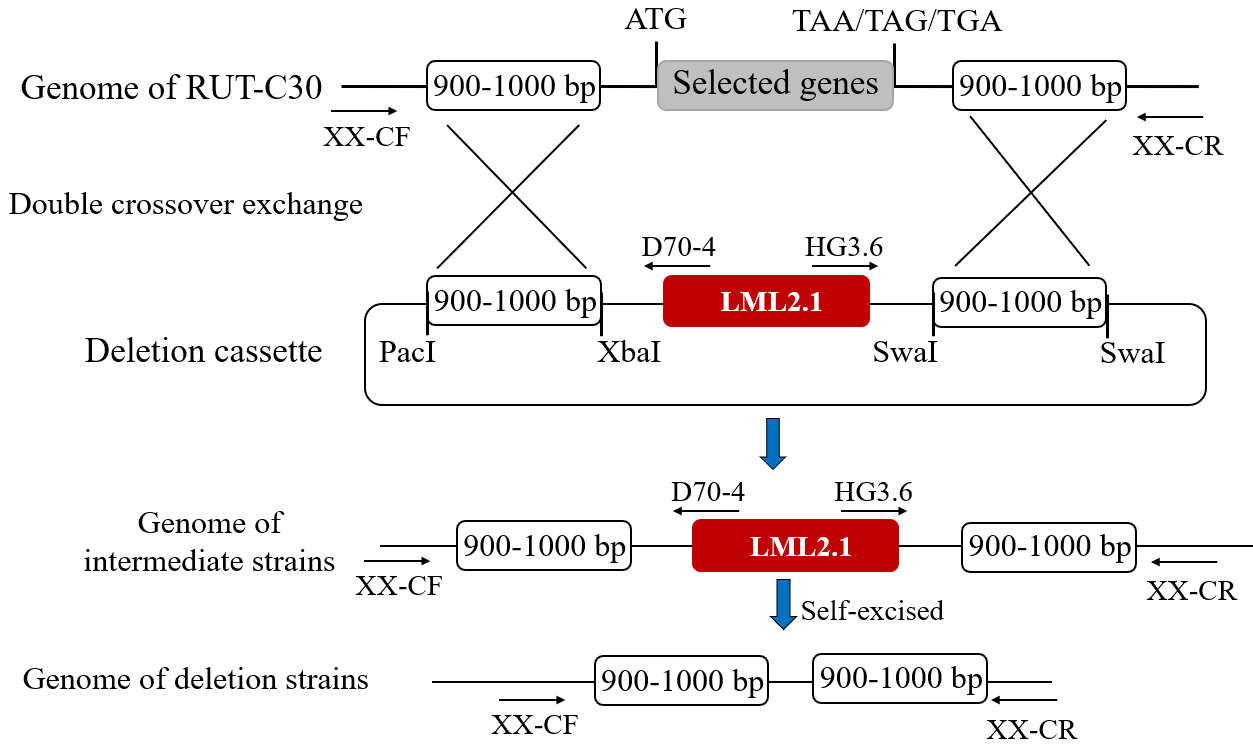


**Fig. S6**
